# Long-Term Benefits of Intermunicipal Cooperation for Small Municipalities in Waste Management Provision

**DOI:** 10.3390/ijerph18041449

**Published:** 2021-02-04

**Authors:** Michal Struk, Eduard Bakoš

**Affiliations:** Faculty of Economics and Administration, Masaryk University, 60200 Brno, Czech Republic; Eduard.Bakos@econ.muni.cz

**Keywords:** municipal solid waste management, intermunicipal cooperation, IMC

## Abstract

Intermunicipal cooperation offers an interesting alternative in cases when municipalities are too small to individually provide public services at an efficient cost level but are reluctant to form a municipal amalgamation in order to benefit from economies of scale. Forming a body consisting of multiple municipalities with a specific focus provides a way to reduce costs on service provision while maintaining municipal sovereignty in other areas. In our paper, we quantify the cost benefits of utilizing intermunicipal cooperation in the field of municipal solid waste management. We examine this using data from a 10-year period from municipalities in the South Moravian Region in the Czech Republic, where high municipal fragmentation results in many dominantly small municipalities that often are not able to provide public services at reasonable costs. This analysis contributes to the literature by conducting a long-term study of the effects of intermunicipal cooperation on public service provision costs. Our results suggest that municipalities participating in intermunicipal cooperation focused on waste management experienced annual cost savings of approximately 13.5% for provision of this service throughout the examined period of 2010–2019 when compared to municipalities that did not cooperate. These long-term results show how beneficial intermunicipal cooperation can be in reducing service costs. In addition to the direct financial benefits, municipal representatives stated that intermunicipal cooperation often brings other qualitative and non-financial benefits such as better service quality, the possibility to share infrastructure, and relief from administrative and managerial burdens through the utilization of professional management, which was especially appreciated by the smallest municipalities with limited administrative staff.

## 1. Introduction

Provision of public services is by its nature a common target of questions regarding costs and efficiency. As public services are financed from public money, the pressure to use these funds appropriately is understandable. The costs of public services become even more important when the municipalities that provide them are very small and thus have problems reaching the optimal size for the efficient provision of such services, or in other words the municipal boundaries are “suboptimal” regarding the provision of local public services [1]. Here we note that there is no strict definition of what a “small” municipality is, and in various countries it could be understood differently. Therefore, for the purpose of this paper we consider a small municipality to be in the range of up to a few thousand inhabitants, but mainly including municipalities with just several hundred inhabitants. The common issue for such small municipalities lies in the fact that the municipality on one hand must provide certain services (often as specifically required by law), but on the other hand due to its small size often cannot provide them at the desired qualitative level or for reasonable per unit costs that are at least close to those of larger municipalities where transaction costs tend to increase much slower with municipality size and economies of scale can be exploited. One way to overcome this issue is to engage in cooperation among multiple municipalities, resulting in a larger coordinated body. By doing so, economies of scale can usually be exploited or, due to the increased size and subsequent negotiating power, better arrangements with the external service provider can be reached. Such arrangements are commonly referred to as intermunicipal cooperation (IMC). The general idea of this type of cooperation was suggested already in the 1960s [2], so IMC itself does not represent a novel idea, but at least in our opinion it is in many suitable cases greatly underused, often due to the lack of convincing evidence on how beneficial IMC can actually be. Therefore, we decided to explore this issue in more detail and contribute to the existing body of empirical literature.

In this paper, we provide an empirical study focused on one typical public service—municipal solid waste management (MSWM)—using data from municipalities in the Czech Republic. The reason MSWM was chosen is because it can be clearly linked with a specific municipality in terms of both inputs and outputs and because this service is in many aspects standardized regardless of municipality, thus making it a good candidate to compare how municipalities deal with this requirement. MSWM itself represents a service that is commonly provided by the public sector (depending on the municipality, either in-house, co-produced, or outsourced) using the money raised from taxes or fees. In the Czech Republic, the responsibility for MSWM is transferred all the way down to individual municipalities and the Czech Waste Act defines the municipality as the body responsible for securing proper waste management (WM). This by itself does not necessarily represent an issue, but municipalities in the Czech Republic are very fragmented and every single one of them bears this responsibility, which requires municipal authorities to become acquainted with the issue at a sufficient level. This becomes a problem in very small municipalities (with sometimes only a few hundred people or even less) where the mayor neither has sufficient time nor can afford additional municipal staff to properly deal with this issue. Such small municipalities then commonly become dependent on whichever WM company is willing to provide WM services but, as the municipality is small, these companies often take advantage of the situation and charge a premium. An individual small municipality simply does not have sufficient negotiating power and does not represent an important enough customer to secure better conditions and better prices. If municipalities are able to act together, however, they can form a larger body that can afford to hire proper specialized management for the entire group and also represents an important customer for WM companies. As a result, they often can secure better service quality and better prices. IMC here represents a way to form such a larger body in a designated area (WM in our case) while still allowing municipalities to maintain their sovereignty in other areas. In the case of the Czech Republic, almost all municipalities charge their citizens annual WM fees and then use them to finance their MSWM. In practice, however, the majority of municipalities find themselves in the situation where the collected fees do not fully cover the costs of MSWM, while the municipalities are still required to ensure this service at a certain level. The reasons for this revenue–cost discrepancy are both legislative (setting the maximum fee that a municipality can charge) and political (people’s general aversion to any tax increase and thus political unwillingness to implement one). Municipalities are then often left with only two options—cost reductions by adjusting contracts with service providers or higher levels of co-financing from public budgets—with both of these options being often significantly difficult for a single small municipality to execute. One way to overcome this is to cooperate with other municipalities and try to reach a better position for the service provision, in terms of both costs and service quality. Such IMC can occur in many areas of public services and is by no means limited to MSWM.

The effects of IMC in WM and other public services have been previously examined in multiple studies, for instance a study examining the use of IMC as a tool for small municipalities to deliver local public services with findings of positive effects from IMCs on costs [3]. A subsequent study [4] concluded that IMC might be a suitable organizational form for some municipalities when providing certain public services, including also positive effects in terms of service frequency and quality [5]. Another study [6], focused specifically on MSWM among Spanish municipalities, concluded that IMC is a pragmatic choice for small municipalities, and, according to [7], this (at least in Spain) applies to medium-sized municipalities as well. Through cooperation, such municipalities are much more likely to exploit economies of scale and thus reduce transaction costs [8]. Positive effects from IMC have also been identified in the wastewater sector [9], especially for small municipalities that can use IMC to overcome municipal fragmentation [10]. In addition to the commonly examined MSWM, positive effects from IMC on reducing municipal spending on public health and fire protection were shown in Japan [11], and similar positive effects occurred also in Brazil with spending on culture, housing, social assistance, and again health care [12]. Within the aforementioned papers, however, in most cases the analysis of IMC benefits was limited to a rather short period (often a few years or even just a single year), making the results partially questionable regarding the long-term sustainability of the observed benefits. This is also an area that we decided to focus on with our study and use a much longer period to examine whether the observed benefits of IMC tend to persist over time.

Another issue strongly related to IMC is municipal fragmentation, which results in a small average size for individual municipalities, often preventing them from reaching efficient levels of public service provision, in terms of both quality and costs. As concluded in a comparison of The Netherlands and Spain, the municipal structure in the country can play an important role in how public services are provided [13]. Spain with smaller municipalities seemed to focus more on consolidation and cooperation, while The Netherlands with generally much larger municipalities seemed to focus more on competition, as the municipalities were already likely large enough to further exploit any economies of scale.

The question of whether to privatize MSWM as a solution to high costs has also been tackled, but the evidence has shown that privatization either has not resulted in consistent savings or that any savings has tended to erode quickly in a few years, while in contrast participation in IMC has seemed to result in systematic savings for the municipality [14,15]. This has also been examined using municipal data from The Netherlands [16], with the conclusion that municipalities that privatized their MSWM experienced savings of 3.5% compared to those with in-house MSWM provision, while municipalities with IMC experienced 4% savings compared to municipalities without IMC. In comparable findings, participation in municipal union as analyzed using a sample of Italian municipalities resulted in MSWM with 5% savings [17]. In addition to cost savings, professional management is also mentioned as an important benefit of IMC, especially for small municipalities [18], together with positive effects from IMC in terms of service cost reduction and overall higher quality of public services [19]. This is also case for the Czech Republic, where mayors of small municipalities (who commonly hold this position only part-time) and their limited administrative staff sometimes find it difficult to meet even basic legislative requirements and have little capacity to focus on additional service improvements or methods of cost reduction. Such a lack of competences, high administrative costs, and resultant logistical inefficiencies seem to be commonly identified as important issues for MSWM in small and rural municipalities [20].

However, there is no unified perspective on the benefits of IMC, and mixed results have also been reported [21,22] with no clear consensus [23]. This observed inconsistency is supported by an analysis of the effects of IMC regarding the costs of 12 common local government services conducted using data from New York State [1], where some of the services showed decreased costs, some did not show any difference in costs, and some even showed an increase in costs. Additional evidence suggests that this was not an isolated case [24].

Studies of this issue are also available for the Czech Republic, namely a study focused on differences between types of IMC institutional arrangements [25], a study focused on the limits of public–private partnerships and contracting out [26], or a study focused on competition and efficiency in MSWM services [27], which again concluded that small municipalities in particular benefit from participating in IMC. A recent metaregression analysis of evidence on IMC benefits in public service provision supports this as well [23].

One might ask why basically all of the aforementioned studies examined the effects of IMC on small municipalities. The answer is straightforward—IMC rather rarely occurs among larger municipalities as they are simply large enough to fully exploit the optimal unit size for public service provision, and therefore have little interest in participating in such cooperation because they would typically gain little benefit from it, if any at all. Why engage in cooperation with other entities if you are able to ensure the service and manage it at an appropriate level on your own? In such cases, service provision would likely only become more complicated due to increased transaction costs. Empirical results supporting this idea did not find significant advantages when a larger municipality took part in IMC [19]. Low interest in IMC among larger municipalities was also observed in Spain [10]. However, for fragmented (and small) municipalities, like those in the Czech Republic, participating in IMC often represents a rational and economic choice that can be explained through transaction costs [28].

The situation in the Czech Republic is to a certain extent specific also for other reasons—as a post-communist country, the Czech Republic has an institutional environment that has undergone a major change since the fall of the previous regime, and regaining of autonomy has led local governments to municipal fragmentation that is among the highest in Europe, with an average municipality size of slightly above 1700 inhabitants (comparable only with France, Slovakia, and Cyprus) and a median of just around 440 inhabitants, with more than 6250 municipalities in total (for comparison, the average municipality in Spain has over 5000 inhabitants, in Italy over 7000, and in The Netherlands over 40,000). This means that the Czech Republic consists dominantly of very small municipalities that commonly face the issue of how to provide a relatively wide range of public services with limited staff and finances resulting from their very small size. Many municipalities, therefore, try to fight their sub-optimal size by utilizing IMC in order to increase the efficiency and effectiveness of public service provision. In the entire Czech Republic, only 6 municipalities (0.1%) have more than 100,000 inhabitants and only 61 municipalities (1%) have more than 20,000 inhabitants. The population density in the Czech Republic is only around 130 inhabitants/km^2^ (and without the three largest municipalities, the value falls to 110 inhabitants/km^2^), which can be considered to be relatively rural. On the other hand, the very high fragmentation also means that municipalities in the Czech Republic are usually not physically very far from each other, which creates a good basis for examining IMC as there are no significant barriers in terms of communication among municipalities, at least from the perspective of distance.

Our goal in this paper is to contribute to the existing empirical literature about the effect of IMC on public spending by quantifying the effects of IMC participation on municipal expenditures using the example of the common public service of MSWM. The questions to be answered are whether IMC provides consistent financial benefits over a longer time period and specifically how much savings can result from utilizing IMC. We analyzed panel data over a 10-year time period from a group of over 600 municipalities from the South Moravian Region in the Czech Republic representing a group of small and very fragmented municipalities, unlike in the majority of previous studies examining this issue. Our analysis thus answers the assertion of several studies that empirical evidence on how IMC affects public spending is still not sufficient [15,29].

## 2. Material and Methods

The data analyzed in this paper consist of municipal population data, municipal expenditure data, and information about the presence of IMC focused on WM in the analyzed group of municipalities, as the public service of WM was selected for the empirical analysis of the effects of IMC. We have chosen to include annual data from 2010–2019 based on their public availability.

Specifically, we used data about municipalities from the South Moravian Region in the Czech Republic collected from the Czech Statistical Office [30]. The Czech Republic has 13 regions (administrative units of a size at the statistical level NUTS 3) plus the capital city of Prague. The total number of municipalities in the South Moravian Region during the analyzed period was 673 (out of 6258 in the entire Czech Republic), with a total area of 7188 km^2^ (out of 78,870 km^2^) and population of 1.19 million (out of 10.69 million at the end of 2019). Compared to the other regions in the Czech Republic, the South Moravian Region is the 4th largest in terms of area and population, 3rd in the number of municipalities, 6th in average municipality size, and 4th in median municipality size. In terms of density, it is above the national average, which is a result of its regional capital of Brno being several times larger than most other regional capitals in the Czech Republic. Brno just by itself accounts for almost one-third of the entire region’s population and thus significantly increases the overall population density of the region. If Brno is excluded, the regional density drops to values comparable with other Czech regions.

However, in the actual analysis we have removed several municipalities from the group for the following two reasons. First, after examining the available data on individual municipalities in the South Moravian Region we have omitted in total three municipalities (including one military area) for which expenditure data regarding MSWM either were not available for the analyzed period or had extremely low values, likely due to misreporting. Second, we have excluded the largest municipalities in the region with populations over 20,000 (6 municipalities in total) as multiple studies have concluded that economies of scale are exhausted above this level [7,31,32,33,34], with some suggesting even the lower threshold of 10,000 [5]. As a result of their already optimal size for service provision, such municipalities typically have very little tendency to cooperate in any way in this area. This in fact proved true as none of these six largest municipalities in the region took part in any IMC body that was included in our analysis. These adjustments left us with 664 municipalities, representing 90% of the total area, 57% of the total population, and 99% of the municipalities in the region. The main difference is in the covered population, which is natural as we deliberately omitted the six largest municipalities.

Table 1 shows selected information about the entire South Moravian Region and information about the analyzed group of municipalities after omitting those without reliable data and those with populations over 20,000. The analyzed group of municipalities thus represents all municipalities from the region that provided the necessary data and that are not too large to exploit the benefits from participating in IMC according to the literature.

If we take into account the strong prevalence of very small municipalities and only the handful of larger municipalities, we can consider the South Moravian Region as quite rural. While the population density of the entire region is around 165 people/km^2^, after excluding the six municipalities larger than 20,000 people the population density of the region drops down to just a little over 100 people/km^2^, which is close to the density values for the entire Czech Republic (136 people/km^2^ including large cities, 83 people/km^2^ without municipalities over 20,000). From this perspective, except for several large municipalities and their surrounding areas, basically the entire Czech Republic can be considered as relatively rural, consisting of many small municipalities scattered throughout the country.

The second group of data, municipal expenditures, was acquired from a public database run by the Czech Ministry of Finance called MONITOR [35]. This database provides public access to, among other things, annual budgetary data for all Czech municipalities, representing a very exhaustive source of information regarding municipal finances in the Czech Republic. In this paper, we focus specifically on expenditures related to MSWM, which is, according to the Czech budgetary structure, represented by the identifiers 3722 (collection and transport of municipal solid waste) and 3725 (treatment of municipal solid waste). We stress here that we focused only on current expenditures that include regular day-to-day expenditures related to WM and not capital expenditures (such as purchasing infrastructure for waste collection) that in practice occur irregularly and are, therefore, not appropriate for intermunicipal comparison of public services provided on a regular basis such as WM.

Collected municipal population data were used together with expenditure data to calculate the average per capita expenditures for MSWM for individual years. Such a step was necessary in order to avoid problems arising from comparing municipalities of notably different sizes. Using per capita values instead of absolute amounts is a common way to offset this issue.

The third type of data used in this paper is whether a given municipality participated in IMC (focused in our case on MSWM). We use the term IMC in this paper for any intermunicipal associations that are legally based and have a focus on WM. In the Czech conditions, these typically include voluntary municipal associations (VAMs), also known as “microregions”. Other, less common forms of IMC include bi/multilateral agreements among municipalities and hybrid organizations for ensuring some specific services (for instance, a WM company owned by municipalities). We did not include these latter specific forms of IMC in our study, as in first case there was practically no available aggregated information on which municipalities used this kind of agreement, and in the second case hybrid organizations are a quite specific type of cooperation that is often not comparable with more common forms of IMC.

Data about municipalities utilizing IMC were acquired from the portal of Regional Information Service [36], which provides a large amount of information about individual municipalities in the Czech Republic, such as municipal infrastructure, selected demographic data, and their participation in IMCs. This portal also contains lists of IMCs with information about the primary focus of the IMC and participating municipalities. From these, we have selected only those IMCs that have specifically stated WM as their focus. In our data set, we used information about participation in IMC as a binary dummy variable. Here we must note that it is possible that some municipalities joined or left such IMCs during the analyzed period, but we expect very few municipalities actually did so, as such arrangements are typically very stable, and additionally there is no database of IMCs with their focus for individual years, so this portal is the best available data source.

In our case (the South Moravian Region), we have identified 5 VAMs/microregions that span from 3 to 42 members (municipalities) with MSWM explicitly listed as the area of their cooperation. In terms of total population, these IMC bodies range from 3000 to almost 55,000—actually meeting the upper proposed limit for efficient WM provision through IMC [37], suggesting that all of these IMC bodies should be able to benefit from efficiency gains from participating in an IMC.

**Figure 1:**
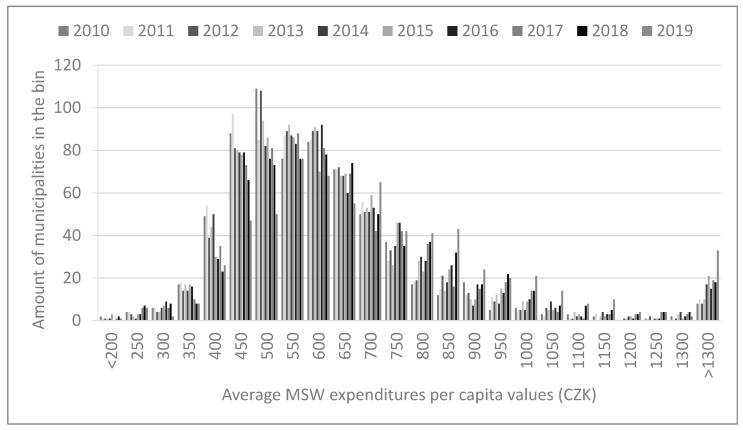
Histogram of average municipal solid waste expenditures per capita in the analyzed group of municipalities, 664 units. Municipalities with reported values over 1300 are grouped in the category >1300.

Figure 1 shows a histogram of per capita current expenditures on MSWM for the municipalities in the analyzed group with values for each of the 10 included periods. The histogram shows the occurrence frequency of certain values or a range of values within a sample. This provides a comprehensive picture of what the most common values are in the sample. As several municipalities occasionally reported relatively high values compared to the majority of the group, in order to preserve the legibility of the histogram, these municipalities are reported together in the category “over 1300”.

From the histogram, we can see that vast majority of municipalities had average per capita MSWM expenditures of CZK 400–850 with a median of CZK 564 and an average of CZK 623 (CZK—Czech koruna; 1 EUR ≅ 25 CZK). There are some extreme values with a couple of municipalities reporting practically no expenditures and several municipalities reporting values several times higher than average. The reasons for such extreme values are, in our experience, mostly misreporting, where the responsible employee entered these expenditures in an incorrect category according to the budgetary structure. There might be a few exceptions where a municipality could truly have very low expenditures, for instance when the municipality has a special agreement with a WM company in exchange for allowing this company to have a landfill within the territory of the municipality, resulting in basically free provision of MSWM services to this municipality, unlike for the company’s other clients, but in practice such cases are very rare and do not notably distort the results of larger samples, such as ours.

In the opposite case, when municipalities report extremely high MSWM expenditures with respect to their size, it is likely a result of incorrect inclusion of capital expenditures on MSWM within the current expenditures category. In this paper, we are examining only current expenditures as these represent expenditures for the municipalities’ regular day-to-day activities and make sense for comparison, unlike in the case of capital expenditures, which occur irregularly and are typically very different between individual years even for a single municipality. Thus, a clear distinction between capital and current expenditures (for instance, purchasing equipment for waste separation or waste collection that has a long lifetime and thus naturally happens only occasionally) is important and necessary.

In some cases, current expenditures might be higher than usual values because the municipality is very remote or has some other specific conditions due to which WM companies agree to provide WM services only with a significant premium, but even in such cases it is hard to justify per capita prices higher than, let us say, three times the usual values, as at such a point it actually becomes economical for the municipality to provide MSWM in-house, even despite high per unit costs compared to the average values for other municipalities. Nevertheless, as there was no simple way to check the true reasons behind the higher expenditures, we included even municipalities with such values in our analysis.

Table 2 contains information about the usage of IMC among the analyzed group of municipalities together with per capita expenditures. We can see that municipalities participating in IMC focused on WM represented 14.0% of the entire group. Neither the average nor median populations of the municipalities changed notably over the analyzed period of 10 years and they gradually increased in total by approximately 5% and 7%, respectively. On the other hand, both average and median WM expenditures per capita showed a much greater increase of approximately 20% over the period, likely caused by inflation of prices over the examined period.

**Figure 2:**
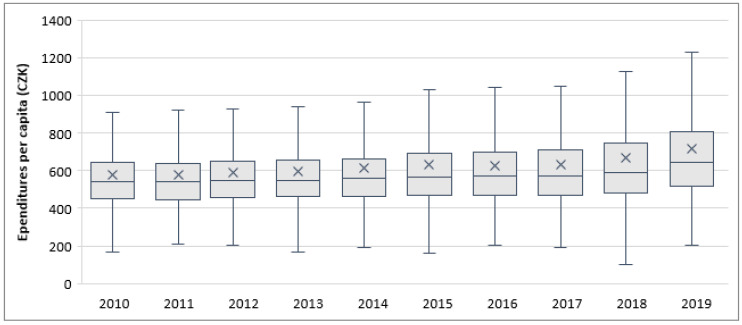
Boxplots of MSWM expenditures per capita during 2010–2019, 664 municipalities.

Figure 2 shows boxplots of per capita WM expenditures in the analyzed group of municipalities for individual years within the analyzed period. In addition to the information from Table 2, we can see that the median values were relatively stable until 2017 (ranging within CZK 540–570 per capita) and only then began to increase more. The standard deviation of per capita expenditures for the period was 317 (ranging within 250–400 for individual years), while the mean value was 623 (ranging within 600–700). Together, these result in the coefficient of variation being 0.51 (ranging within 0.45–0.55), which means that the variation in the observed data can be considered low. This was to be expected, as MSWM represents a relatively unified service and, with WM companies competing among them, in general there should not be very large differences among the relative expenditures of individual municipalities unless some specific conditions are present.

For the subsequent analysis of the expenditures, we used two methods. The first is a statistical analysis consisting of comparing the calculated per capita expenditures in individual years and differentiating between municipalities participating in IMC focused on WM and municipalities that did not participate in any kind of IMC focused on this field (although these municipalities may have participated in IMCs focused on other areas). When larger samples are used, such as in our case, calculating these values provides very clear and straightforward information about the development of the variables and can be directly and easily interpreted in terms of probable effects from IMC on the results.

Second, we performed an econometric analysis of the per capita expenditures. We conducted standard ordinary least squares (OLS) regression using the acquired panel data. This statistical method basically estimates the quantitative role of each included independent variable in calculating the value of the chosen dependent variable—or simply put, how the value of the dependent variable is constructed from the available independent variables. It represents a type of least squares method for estimating parameters in a linear regression model that could have an impact on the dependent variable (in our case per capita expenditures). In our case, we do not expect more complex relations than linear to occur, and thus OLS is an appropriate estimating method. Moreover, compared to other more complex methods, interpretation of linear regression is relatively straightforward as it estimates the linear function with the smallest squared differences between the observed values of the dependent variables, or in other words the linear function with the best fit to the examined data. The expected result of such an analysis should be basically a function of the average per capita expenditures consisting of the effects of the individual examined independent variables. OLS represents a standard econometric method commonly used for data analysis and more detailed explanation of the method can be found in numerous econometric and statistical textbooks.

Overall, the method of the conducted analysis can be summarized as a process consisting of the following subsequent steps:

(1) Collection of data on municipal expenditures, municipal populations, and presence of IMC related to MSWM provision for the selected group of municipalities;
(2) Calculation of per capita values of MSWM expenditures and dividing municipalities into groups based on IMC presence;
(3) Comparison of calculated differences in waste-related expenditures between municipalities with and without IMC;
(4) Regression analysis focused on the effects of IMC presence on waste-related expenditures;
(5) Interpretation of the results and discussion with the available literature on the issue.

## 3. Results and Discussion

In this part, we provide the results of the empirical analysis of the acquired data together with a discussion of the results and reflection on them with the available theory regarding the examined issue.

**Figure 3:**
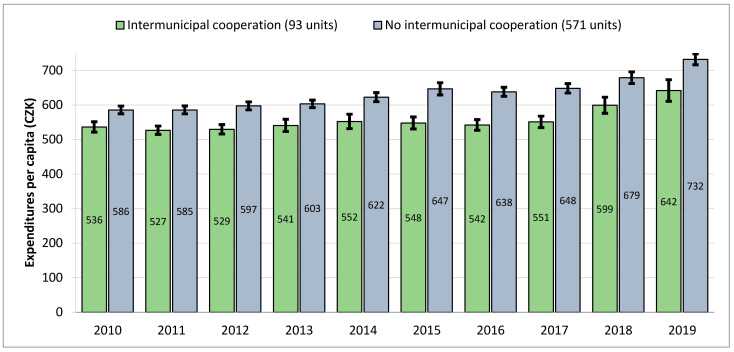
Average MSWM expenditures per capita during 2010–2019, 664 municipalities, standard error of the mean (SEM) bars included.

Figure 3 shows the average per capita expenditures on MSWM for each year from the examined 10-year period, distinguishing between municipalities utilizing IMC and those without IMC. The results show consistent differences. Municipalities utilizing IMC had lower annual per capita expenditures in the individual analyzed years, with values between 9% and 18% and an average of 13.8%. In the case of the median values (not shown in Figure 3), the annual savings were between 5% and 12% with an average of 8.6%. While such differences might not seem so high at first glance, we should keep in mind that these savings occurred consistently throughout a long period and gradually added up. In particular, in the cases of the very small municipalities where, according to our calculations using financial data for Czech municipalities, MSWM expenditures can account for up to 10% of the total annual budget, such differences can result in savings of over 1% of the annual budget just by improving MSWM provision.

As was partially shown in Table 2, per capita expenditures tended to increase over time, and Figure 3 shows this in more detail. We see that in 2010 average per capita expenditures in those municipalities not participating in IMC were more than 9% higher, while this difference increased throughout the period up to 14% (in addition to the overall cost increase in MSWM by 20% during the period). This increasing difference between the cost for municipalities participating in IMC and the cost for those that did not participate makes IMC an even more appealing alternative compared to ensuring services individually. In the case of median values of per capita expenditures (not shown), the trend was similar, with the difference spanning from 6% to 12%.

For a more thorough statistical analysis, we performed an OLS regression on the pooled per capita expenditure data including the entire analyzed period. By using OLS regression, we were able to estimate the effect of considered independent variables (IMC presence and period) on the dependent variable (per capita expenditures). We have created two models, with the first including only the IMC participation variable, and the second including both IMC participation and a time-related variable representing different years. In both cases, we also created models with robust standard errors. The regression analysis provides us with the precise estimation of the monetary effects of IMC that can be used directly as supporting empirical evidence when considering the utilization of IMC. The outcomes of the regression are presented in Table 3.

Model 1 suggests that presence of IMC reduced average per capita expenditures by CZK 77 (12.1%) from the initial CZK 634 represented by a constant. Both values in the model are statistically significant (at the 1% level).

Model 2 expands on the first model and again suggests that the presence of IMC reduced average per capita expenditures by CZK 77 (13.5%) from the initial CZK 572 represented by a constant, while each consecutive year increased this total per capita value by an additional CZK 13.6 (2.4%). This basically says that average per capita expenditures on MSWM increased each year by 2.4%, representing something like the inflation of per capita MSWM expenditures throughout the period. Again, all values in the model are statistically significant.

Formally, Model 1 can be written as

$$avgexp_{pc}=633.68-77.00*IMC+\varepsilon$$

where

*avgexp_pc_* stands for average expenditures per capita on MSWM,
*IMC* stands for the municipality participating in IMC focused on WM (binary variable), and
*ε* stands for random error.

This says basically that a municipality without IMC is likely to have average per capita expenditures on MSWM of CZK 634, while a municipality with IMC would have per capita expenditures of CZK 557. While such a difference might not seem so high, we should keep in mind that these annual savings of 12.1% add up over time.

Next, Model 2 can be written as:

$$avgexp_{pc}=572.50-77.00*IMC+13.60*period+\varepsilon$$

where,

*avgexp_pc_* stands for average expenditures per capita on MSWM,
*IMC* stands for the municipality participating in IMC focused on WM (binary variable),
*period* stands for the period for which the model is calculating the result (0 for 2010, 1 for 2011, 2 for 2012, and so on), and
*ε* stands for random error.

As in the previous case with Model 1, this result can be interpreted as stating that a municipality without IMC would have had average per capita expenditures of CZK 572 in 2010 with annual increases by CZK 13.6 for each consecutive year, while in the case of a municipality with IMC the average per capita expenditures would have been CZK 496 in 2010 again with annual increases by CZK 13.6 for each consecutive year in the analyzed period. As stated above, utilizing IMC in this model resulted in annual savings of 13.5%, which is consistent with the observation from Figure 3 that the expenditure difference between municipalities with and without IMC tended to increase over time. Compared to the interpretation of Model 1, the added information about the annual increase in expenditures (or the estimated effect of the period variable) can be viewed as information on how long it would take a municipality utilizing IMC to reach the expenditure levels of municipalities without IMC. In this specific case, ceteris paribus, a municipality without IMC is approximately 5 years ahead in expenditure levels, or in other words it would take an additional 5 years for a municipality using IMC to reach the present average expenditure levels of a non-cooperating municipality.

Regarding the provided model statistics we note that although the values of adjusted R^2^ (the coefficient of determination) in our models were relatively small, in this case it is not an issue as our goal was not to precisely identify which components account for total expenditure, but to examine the effects of selected factors (independent variables) on the selected dependent variable. What our results say is that the examined variables are statistically significant, but there are additional factors that affect individual municipal expenditures that are not included in our analysis. These factors may include, for instance, distance to the closest final waste treatment facility (increased transportation costs), number of WM companies willing to provide MSWM (level of competition), and the requirements of the municipality about what specifically the MSWM service should include (such as frequency of collection, specific types of waste-collecting vehicles, etc.). Inclusion of such variables would likely increase the overall explanatory power of the model and make it possible to estimate more precisely the expenditure levels of individual municipalities. However, acquiring such detailed data is practically impossible considering the variability of potentially relevant municipal characteristics and their individual uniqueness.

The results of our models thus suggest that IMC in MSWM provided annual cost reductions of approximately 13.5%. Such results are in general accordance with a 4% cost reduction observed in The Netherlands [16], a 5% cost reduction in Italy [17], and an up-to-10% cost reduction calculated in the Czech Republic [24], depending on the form of IMC. In our case, however, the estimated reductions are notably higher than those in The Netherlands and Italy, which might have been caused by the fact that municipalities in the Czech Republic are on average much smaller in population size than those in The Netherlands and Italy, supporting the observation that IMC tends to yield greater benefits the smaller the municipalities taking part in it are [10,19,26].

Although annual cost reductions of 13.5% might not seem so high, for many municipalities any kinds of saving matter. The importance of savings becomes even more apparent when longer periods are considered. In our case, a 10-year period was included. If summed up, the total savings according to the models could reach one full year of MSWM expenditures in approximately 7–8 years, which can represent quite a considerable sum of money that can be used for important municipal investments, or alternatively, the municipality can decrease local fees for WM, which is always appreciated by the public and can be notably beneficial from a political perspective.

Nevertheless, we find it important to emphasize here that although the results might suggest that IMC is a straightforward tool for cost reduction, it does not work automatically, and the results are not guaranteed. In territorially consolidated countries with larger units, IMC is prone to yielding only marginal benefits, if any [38]. At the end of the day, it depends to a great extent on the individual public officials and their willingness to engage in functioning IMC and the previous state of MSWM in a given municipality. There are clearly transaction costs that arise when participating in IMC (such as costs related to hiring professional management and costs related to service quality monitoring), but the available evidence shows that these increased transaction costs are usually offset by a decrease in per unit service costs as the serviced area increases (economies of scale), thus resulting in an overall decrease in total costs for the public service [7,39]. However, should the number of members of an IMC body become too large, the internal decision-making process might become a nuisance itself [10].

In addition to the demonstrated empirical advantages to participating in IMC, we see another benefit from this paper in the presented evidence of IMC as an alternative to municipal mergers/amalgamation, which is commonly suggested as a solution to territorial fragmentation [40]. When considering the Czech Republic with its strong opposition to basically any kind of municipal merger, the level of territorial consolidation achieved in some other countries with significant reductions in the number of municipalities remains in the Czech conditions practically unimaginable. This is reflected in the fact that there have been practically no cases of successful amalgamation in recent years. In such a situation, IMC seems to serve as a viable alternative that, according to the results shown in this paper, has the potential to bring consistent financial benefits in the long run.

However, it should be also noted that in addition to such advantages as potentially better allocation of resources, IMC can have certain disadvantages and weaknesses. Negatives can include the weakening of local democracy and a general requirement of additional effort by the local authorities to actually achieve the potential positive results, as otherwise the opposite can occur [38]. The non-monetary price for the expected cost reductions may include a partial (although generally relatively small) loss of sovereignty as by entering into IMC a municipality has to accept certain compromises, follow set rules, and become part of a team. Occasionally, this might be too much for a municipality, especially if there is strong leading municipality or sub-group of municipalities within the IMC that tend to put their own interests up front and in this way try to “exploit the weak”. Nevertheless, such situations usually do not last very long as there is no point in a disagreeing municipality remaining in such an IMC agreement unless the savings still prevail over the negative aspects.

Finally, what has been covered only briefly so far are the non-financial aspects of participating in IMC. According to our informal survey among municipal representatives in Czech municipalities, many benefits of participating in IMC are of a qualitative nature. Once municipalities form a larger body that acts unified in selected areas, they often become much more attractive to companies who had not previously been interested in providing services to these individual small municipalities. This increased “attractiveness” typically results in improved competition that can produce both lower costs and better services for the municipalities. Considering our case with WM, multiple municipal representatives told us that once they took part in IMC, WM companies started to respond better to their requests, decreased their reaction time when additional service was needed, and so on. Such perceived improved quality of public services among municipal representatives after engaging in IMC was also observed using a random sample of small Italian municipalities [19]. Alternatively, for a larger group of municipalities within an IMC body it might even become economical to form a hybrid organization (a mixed public–private company where ownership is divided between the public and private sector while operating fully under private commercial law) for service provision and thus put the service fully under the control of these municipalities and subsequently tailor services with respect to their individual needs. Another perceived benefit of IMC was the possibility of sharing available infrastructure among cooperating municipalities and in this way spreading out the costs for otherwise insufficiently utilized (and as a result costly) infrastructure [26]. Such cooperation can include, for instance, sharing services of civic amenity sites to people from both the original municipality and cooperating municipalities, the possibility to share a biogas or composting plant among a group of municipalities, and so on. Moreover, when IMC seems to work well, municipalities sometimes decide to invest together into new infrastructure that would otherwise not make economic sense for a single municipality due to insufficient utilization of such infrastructure. Some representatives mentioned even another benefit from joining IMC in the possibility to apply for governmental funding that was available only for a certain size of applying entity and thus often unavailable for very small municipalities.

Looking at the preceding from a more general perspective, if municipalities are willing to communicate with each other properly and get along, IMC seems to offer quite broad and significant potential for improvement in public service provision (not limited to just MSWM), especially for small municipalities. As was shown, IMC basically provides an option to artificially create a subject with a size that makes sense in the provision of many public services while still allowing these municipalities to keep their formal independence and sovereignty to basically the full extent. The evidence presented in this paper should contribute to the wider consideration of IMC as a suitable approach to overcome many issues commonly associated with small municipalities.

## 4. Conclusions

Our research focused on the long-term financial effect of IMC in the area of MSWM. An analysis of data from over 600 municipalities in the South Moravian Region in the Czech Republic spanning over a 10-year period showed consistent 13.5% annual savings in MSWM provision for municipalities utilizing IMC in this area. While this might not seem like so much for an individual year, if aggregated for multiple years, total savings for the municipality can become notable and open up possibilities for further improvements in MSWM provision, decreases in collected fees, or simply savings on resources for other alternative uses.

This finding represents the main contribution of this paper as most of the previous studies that focused on the effects of IMC on public spending used only a very limited time-period, while in our case 10 consecutive periods are included. Moreover, very few studies have examined this topic in the conditions of Central and Eastern Europe, and so we aimed to fill this gap as well. From a more general perspective, our study showed how beneficial IMC can be when providing public services—in this case, the example of MSWM was used—and the study also showed that the benefits tended to last. Another important key point to emphasize here is that our analysis was focused on small municipalities, which should be able to benefit the most from utilizing IMC. Therefore, our results can be especially relevant for regions and areas with many scattered small municipalities. On the other hand, where the municipal structure consists dominantly of larger municipalities, the benefits from utilizing IMC are likely to be much lower, if any are present at all.

Our study also presents IMC as an alternative to municipal merging. In order to increase the effectiveness of public spending, cooperation seems to offer a way to both maintain municipal independence and sovereignty and benefit from being a part of a larger body. Such associations of municipalities are often able to secure services for the participating municipalities at better prices through exploiting economies of scale and negotiating better overall deals with external service providers. While participation in an IMC is usually connected with increased transaction costs, benefits from exploiting economies of scale by overcoming sub-optimal municipal size through cooperation tend to offset these costs—better results typically require investing in better tools or procedures first.

In addition to the quantitative benefits of IMC, an informal survey among local representatives suggests the presence also of qualitative benefits in the form of better service quality (such as better response times, higher waste collection frequency, and the provision of additional waste-related services) and a better overall position when negotiating with external service providers once a municipality has joined an association of multiple municipalities. Participating in a larger body of municipalities also offers possibilities for utilizing more professional management which, especially in the case of small municipalities, can bring great relief to the administrative staff. Professionally hired specialized staff focused on MSWM for multiple municipalities are very likely to have better information and generally better knowledge on how to secure MSWM than the typical mayor of a small municipality who has numerous other tasks to complete and usually very limited administrative staff for assistance. While hiring such professional staff requires additional costs, if these costs are split among a group of municipalities, in the end it can actually be cheaper for individual municipalities as it can relieve current staff from dealing with this issue by delegating it to professionals, thus allowing the staff to focus more on other duties. In the case of the Czech Republic, where municipal fragmentation is very high, this represents an important non-financial benefit to municipalities and proper evidence that it works can improve their opinion of participation in some form of IMC. Finding such alternative ways to improve public service provision and decrease costs is important in situations where municipalities seem to be very reluctant to enter into any kind of municipal amalgamation (as is the case not just in the Czech Republic), which could otherwise be used to solve such issues.

## Figures and Tables

**Table 1:** Selected demographic data about the South Moravian Region (CZE), the entire region and the analyzed group of municipalities *, in 2010 and 2019.

Data Set	No. of Municipalities	Area (km^2^)	Total Population		Population Density (per km^2^)	
			2010	2019	2010	2019
Entire region	673	7188	1,163,627	1,191,989	161.9	165.8
Analyzed group *	664	6453	650,294	680,163	100.8	105.4

* Municipalities excluding three municipalities with misreported data and six municipalities with over 20,000 inhabitants.

**Table 2:** Analyzed group of municipalities, distribution of intermunicipal cooperation (IMC) presence, population, average and median municipal solid waste management (MSWM) expenditures per capita, 664 units, selected years 2010–2019.

Period		2010	2013	2016	2019
No. of Analyzed Municipalities		664	664	664	664
With IMC		93	93	93	93
No IMC		571	571	571	571
Average (median) population		979 (554)	993 (569)	1007 (579)	1023 (595)
MSWM expenditures per capita (CZK)	Median	539.5	549.0	570.1	644.9
	Average	578.7	594.3	624.4	719.4

**Table 3:** Regression analysis of IMC presence on MSWM expenditures/capita (CZK), 2010–2019 pooled data, 664 units.

	OLS Model 1	OLS Model 1, Robust (HAC) s.e.	OLS Model 2	OLS Model 2, Robust (HAC) s.e.
Constant	633.68 ***	633.68 ***	572.50 ***	572.50 ***
	(4.17)	(4.39)	(7.31)	(6.51)
IMC presence	−77.00 ***	−77.00 ***	−77.00 ***	−77.00 ***
	(11.15)	(7.53)	(11.07)	(7.46)
Period			13.60 ***	13.60 ***
			(1.34)	(1.34)
No. of observations	6640	6640	6640	6640
*p*-value (F)	0.000	0.000	0.000	0.000
S.D. dependent variable	316.551	316.551	316.551	316.551
S.E. of regression	315.445	315.445	313.041	313.041
Adj. R^2^	0.007	0.007	0.022	0.022

*** stands for statistical significance of the coefficient at 1%, standard errors in parentheses.

## Data Availability

The data presented in this study are available on request from the corresponding author.

## References

[1] Aldag A.M., Warner M.E., Bel G. (2019). It Depends on What You Share: The Elusive Cost Savings from Service Sharing. J. Public Adm. Res. Theory.

[2] Ostrom V., Tiebout C.M., Warren R. (1961). The organization of government in metropolitan areas: A theoretical inquiry. Am. Political Sci. Rev..

[3] Bel G., Fageda X. (2006). Between privatization and intermunicipal cooperation: Small municipalities, scale economies and transaction costs. Urban Public Econ. Rev..

[4] Bel G., Fageda X. (2008). Reforming the local public sector: Economics and politics in privatization of water and solid waste. J. Econ. Policy Reform.

[5] Bel G., Mur M. (2009). Intermunicipal cooperation, privatization and waste management costs: Evidence from rural municipalities. Waste Manag..

[6] Bel G., Fageda X., Mur M. (2014). Does Cooperation Reduce Service Delivery Costs? Evidence from Residential Solid Waste Services. J. Public Adm. Res. Theory.

[7] Pérez-López G., Dollery B., Tran C.D.T.T. (2021). Is Council Cooperation Cost Efficient? An Empirical Analysis of Waste Collection in Spanish Local Government, 2009 to 2015. Public Money Manag..

[8] Bel G., Fageda X., Mur M. (2013). Why Do Municipalities Cooperate to Provide Local Public Services? An Empirical Analysis. Local Gov. Stud..

[9] Blaeschke F., Haug P. (2017). Does intermunicipal cooperation increase efficiency? A conditional metafrontier approach for the Hessian wastewater sector. Local Gov. Stud..

[10] Puey E.P., Ferran J.M., Mussons C.P. (2018). Beyond size: Overcoming fragmentation by inter-municipal associations in Spain? The case of Catalonia. Int. Rev. Adm. Sci..

[11] Baba H., Asami Y. (2019). Municipal population size and the benefits of inter-municipal cooperation: Panel data evidence from Japan. Local Gov. Stud..

[12] Silvestre H.C., Marques R., Dollery B.E., Correia A.M. (2019). Is cooperation cost reducing? An analysis of public–public partnerships and inter-municipal cooperation in Brazilian local government. Local Gov. Stud..

[13] Bel G., Dijkgraaf E., Fageda X., Gradus R. (2010). Similar Problems, Different Solutions: Comparing Refuse Collection in The Netherlands and Spain. Public Adm..

[14] Hefetz A., Warner M.E., Vigoda-Gadot E. (2012). Privatization and Intermunicipal Contracting: The US Local Government Experience 1992–2007. Environ. Plan. C Gov. Policy.

[15] Bel G., Warner M.E. (2015). Inter-Municipal Cooperation and Costs: Expectations and Evidence. Public Adm..

[16] Dijkgraaf E., Gradus R. (2013). Cost advantage cooperations larger than private waste collectors. Appl. Econ. Lett..

[17] Ferraresi M., Migali G., Rizzo L. (2018). Does intermunicipal cooperation promote efficiency gains? Evidence from Italian municipal unions. J. Reg. Sci..

[18] Bel G., Warner M.E. (2015). Factors explaining inter-municipal cooperation in service delivery: A meta-regression analysis. J. Econ. Policy Reform.

[19] Giacomini D., Sancino A., Simonetto A. (2018). The introduction of mandatory inter-municipal cooperation in small municipalities: Preliminary lessons from Italy. Int. J. Public Sect. Manag..

[20] Põldnurk J. (2015). Optimisation of the economic, environmental and administrative efficiency of the municipal waste management model in rural areas. Resour. Conserv. Recycl..

[21] Bel G., Gradus R. (2017). Privatisation, contracting-out and inter-municipal cooperation: New developments in local public service delivery. Local Gov. Stud..

[22] Frère Q., Leprince M., Paty S. (2010). The impact of intermunicipal cooperation on local public spending. Urban Stud..

[23] Bel G., Sebő M. (2021). Does Inter-Municipal Cooperation Really Reduce Delivery Costs? An Empirical Evaluation of the Role of Scale Economies, Transaction Costs, and Governance Arrangements. Urban Aff. Rev..

[24] Allers M.A., De Greef J. (2016). Intermunicipal cooperation, public spending and service levels. Local Gov. Stud..

[25] Soukopová J., Vaceková G. (2018). Internal factors of intermunicipal cooperation: What matters most and why?. Local Gov. Stud..

[26] Soukopová J., Vaceková G., Klimovský D. (2017). Local waste management in the Czech Republic: Limits and merits of public-private partnership and contracting out. Util. Policy.

[27] Pavel J., Slavík J. (2017). The relationship between competition and efficiency of waste-collection services in the Czech Republic. Local Gov. Stud..

[28] Casula M. (2020). A contextual explanation of regional governance in Europe: Insights from inter-municipal cooperation. Public Manag. Rev..

[29] Holzer M., Fry J.C. (2011). Shared Services and Municipal Consolidation: A Critical Analysis.

[30] Czech Statistical Office. www.czso.cz.

[31] Bel G., Costas A. (2006). Do Public Sector Reforms Get Rusty? Local Privatization in Spain. J. Policy Reform.

[32] Callan S.J., Thomas J.M. (2001). Economies of Scale and Scope: A Cost Analysis of Municipal Solid Waste Services. Land Econ..

[33] Holzer M., Fry J., Charbonneau E., Van Ryzin G., Wang T., Burnash E. (2009). Literature Review and Analysis Related to Optimal Municipal Size and Efficiency.

[34] Stevens B.J. (1978). Scale, Market Structure, and the Cost of Refuse Collection. Rev. Econ. Stat..

[35] Monitor—Complete Overview of Public Finance. https://monitor.statnipokladna.cz/.

[36] RIS—Regional Information Service. www.risy.cz.

[37] Sarra A., Mazzocchitti M., Nissi E. (2020). A methodological proposal to determine the optimal levels of inter-municipal cooperation in the organization of solid waste management systems. Waste Manag..

[38] Franzke J., Klimovský D., Pinterič U. (2016). Does Inter-Municipal Cooperation Lead to Territorial Consolidation? A Comparative Analysis of Selected European Cases in Times of Crisis. Local Public Sector Reforms in Times of Crisis.

[39] Baba H., Asami Y. (2019). Estimating the minimal efficient scale and the effect of intermunicipal cooperation on service provision areas for waste treatment in Japan. Asia-Pac. J. Reg. Sci..

[40] Swianiewicz P. (2010). If Territorial Fragmentation is a Problem, is Amalgamation a Solution? An East European Perspective. Local Gov. Stud..

